# Supplementary material for: Fast, Broad-Band Magnetic Resonance Spectroscopy with Diamond Widefield Relaxometry
Source: ACS Sens. 2023 Apr 12;8(4):1667–75. doi: 10.1021/acssensors.2c02809 (PMC10152489; doi:10.1021/acssensors.2c02809)
Supplement: Supplementary file 1 — se2c02809_si_001.pdf [file se2c02809_si_001.pdf]

Supporting Information:

Fast, broad-band magnetic resonance spectroscopy with diamond widefield relaxometry

Charles Mignon[1,#], Ari R. Ortiz Moreno[1,#], Hoda Shirzad[2], Sandeep K. Padamati[1], Viraj G. Damle[1], Yori Ong[1], Romana Schirhagl[1,*], Mayeul Chipaux[1,2,*]

# these authors contributed equally

1. Groningen University, University Medical Center Groningen, Antonius Deusinglaan 1, 9713 AW Groningen, the Netherlands
2. Institute of Physics, École Polytechnique Fédérale de Lausanne (EPFL), CH-1015 Lausanne, Switzerland

* Correspondance: romana.schirhagl@gmail.com mayeul.chipaux@epfl.ch

1. Magnetic field simulation

In order to optimize the position of the cylindrical magnet, we performed a simulation using the expression derived in ([1]), and using the magnetization given by the dipole approximation as the magnetization in the axis of the magnet.

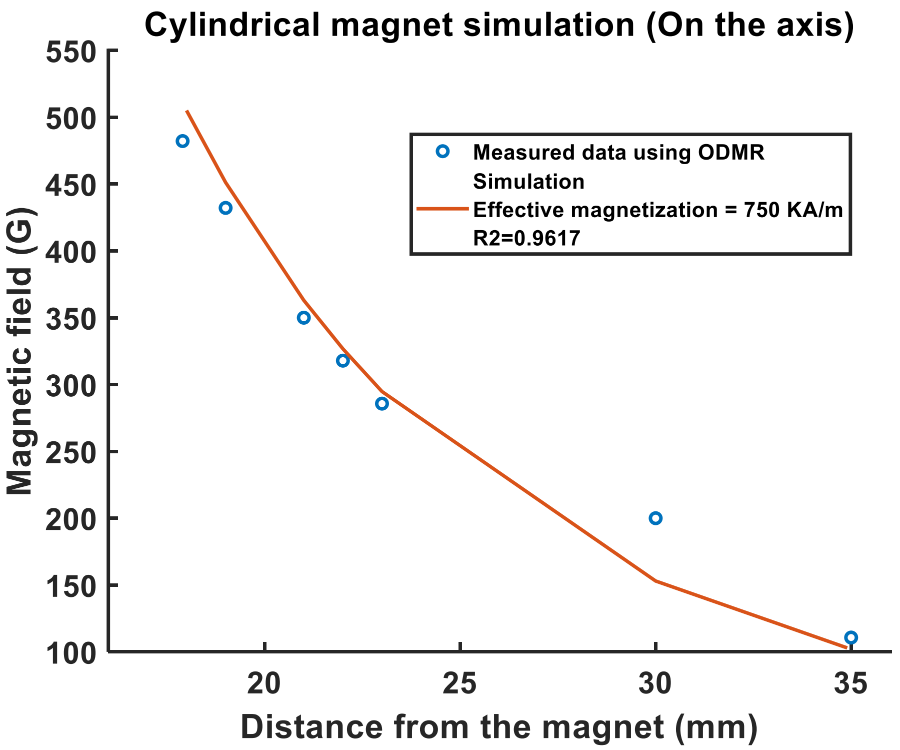

Figure 1S: Simulated and measured magnetic field on the cylindrical magnet axis.

2. X band EPR for the copper complex

We validated our findings by performing a traditional EPR measurement in our sample.

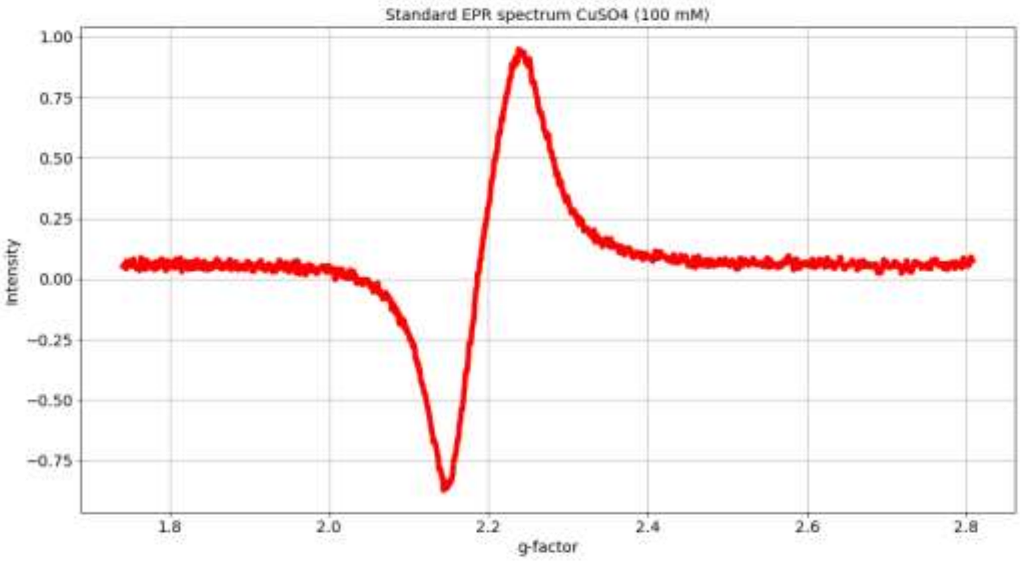

Figure 2S: EPR spectrum of the $CuSO_4$ sample.

3. Magnetic field visualization

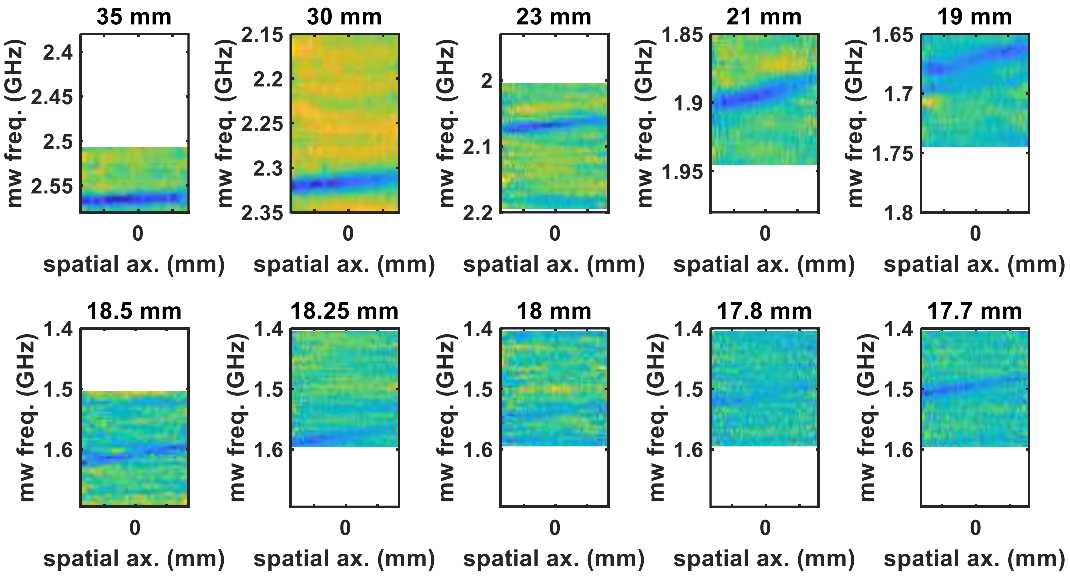

Figure 3S: Microwave photoluminescence response along the diagonal axis (one pixel width) as a function of the microwave frequency

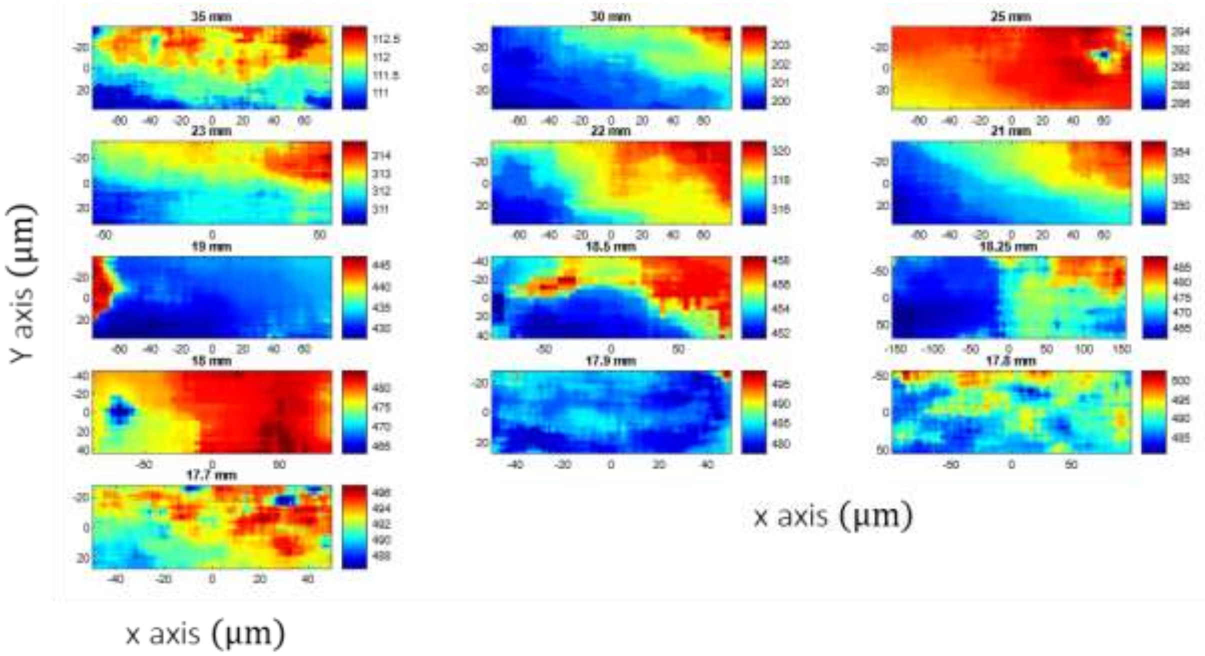

Figure 4S: Magnetic field maps. The values of the magnetic field results from the Lorentzian fit of the microwave response spectrum. Thus, the magnetic field value is the projection of the local magnetic field on the aligned NV-centre direction.

4. Spin relaxation contrast

We also present the average over the whole relaxometry images as a function of the magnet position.

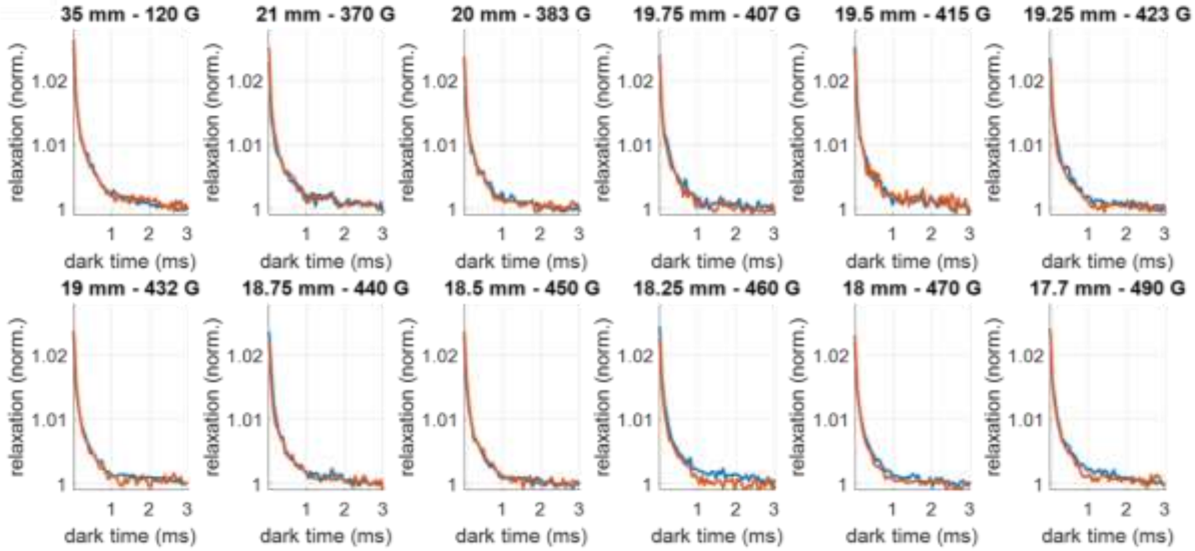

Figure 5S: Microwave-free electron paramagnetic resonance spectroscopy. Surface average T1-relaxation curves in nitric acid solution (1 mM), blue curves, and nitric acid-copper sulfate solution (100 mM CuSO4 dissolved in 1 mM HNO3), red curves at decreasing distances magnet/diamond, i.e. increasing magnetic fields (A). T1-relaxation curves off and on resonance: 35 mm-110 G (left) and 18.1 mm-460 G (right).

5. Subsampled acquisitions

EPR spectra reconstructed from small subset of the entire acquisition. Chosing only two darktimes ($\tau_s = 1$ and $\tau_l = 3$ ms such that $T_\tau = 4$ ms) but keeping all the sequences $N_{seq} = 10$, or even considering only the first sequence ($N_{seq} = 1$). This diminishes the acquisition times T_{ac} from 31 minutes to 1.6 min and 10 s for each position of the magnet. Figure 6 shows the resulting spectra corresponding to the three situations.
We note that the resonant peak at ~460 G is present in all three spectra. Yet, as expected, we observe a cleaner spectrum when using the complete set of darktimes.

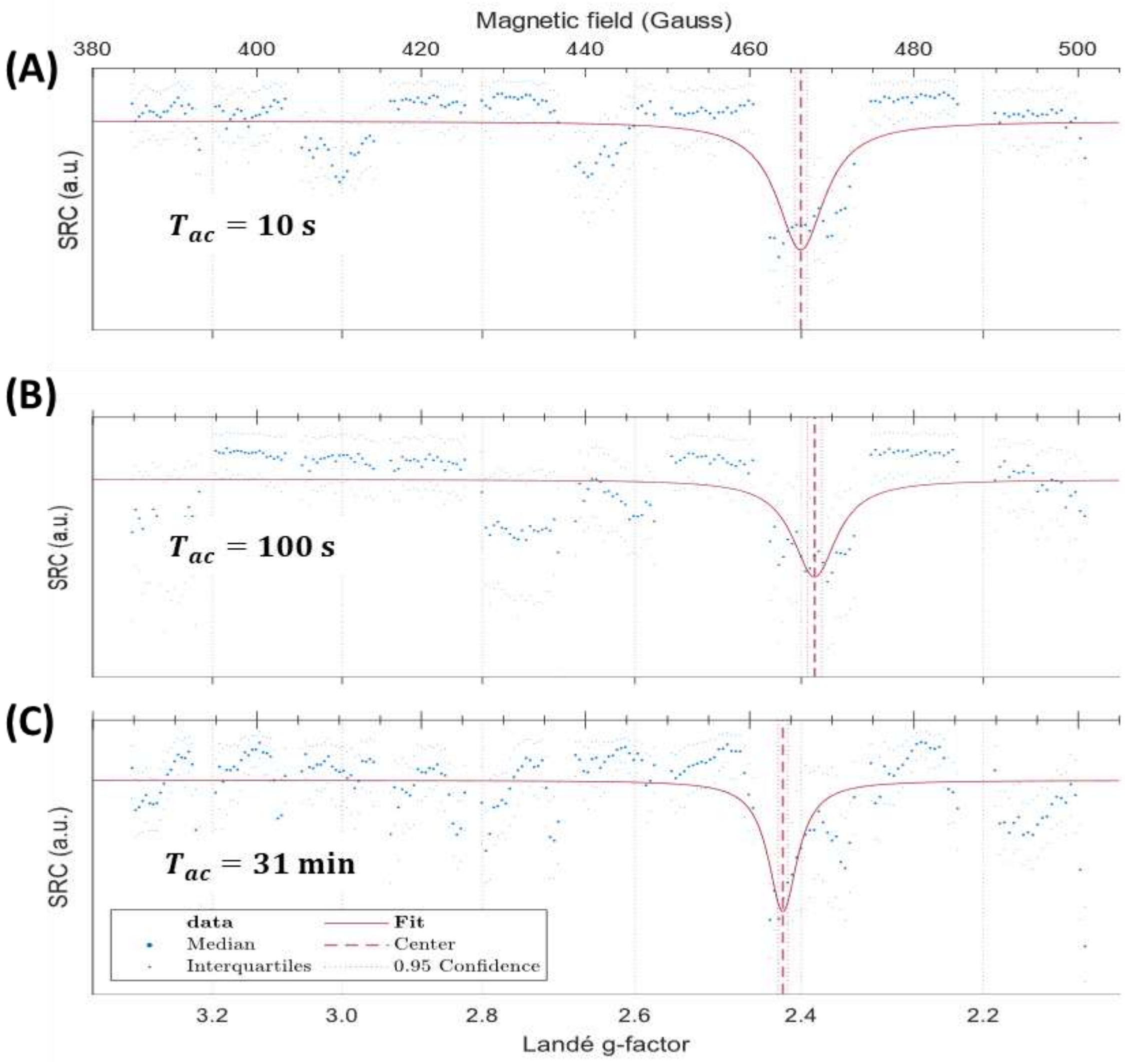

Figure 6S: Electron paramagnetic spectroscopy using subset of the acquired data. (A) EPR spectrum calculated using PL images at two dark times (1 and 3 ms) $T_\tau = 4$ms and only one single repeat ($N_{seq} = 1$), (B) using PL images at two dark times (1 and 3 ms) and $N_{seq} = 10$, and (C) using the entire dataset. (50 dark times for a total of $T_{tau} = 75$ ms in total) and$N_{seq} = 10$. Estimated acquisition times are indicated for a single position of the magnet. (The shown range corresponds to 11 different position out of the 13 acquired.

6. Apparent coherence time

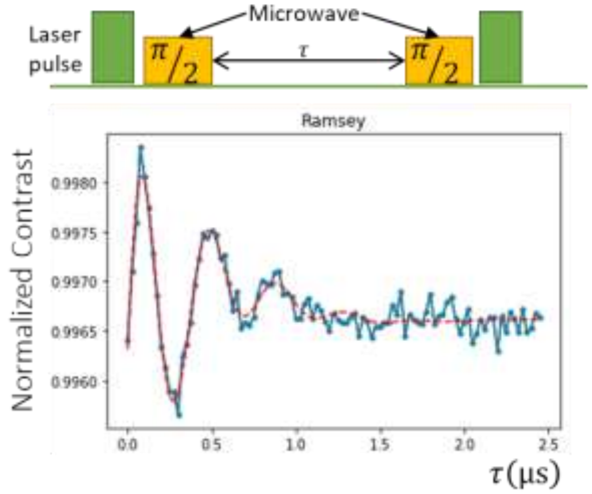

Figure 7S: Ramsey fringes at 1.75GHz

Fit function:

$$y = C_1 \sin(f_1 \tau) \exp\left(-\frac{\tau}{\tau_1}\right) + C_2 \sin(f_2 \tau) \exp\left(-\frac{\tau}{\tau_2}\right)$$

Fitted parameters:

$C_1 = -2 \cdot 10^{-3}$	$C_2 = 2 \cdot 10^{-3}$	
$f_1 = 0.42$	$f_2 = 2.45$	MHz
$\tau_1 = 0.440$	$\tau_2 = 0.330$	μs

7. References

1 Caciagli, A., Baars, R. J., Philipse, A. P., & Kuipers, B. W. (2018). Exact expression for the magnetic field of a finite cylinder with arbitrary uniform magnetization. Journal of Magnetism and Magnetic Materials, 456, 423-432.
